# Controlling one’s world: Identification of sub-regions of primate PFC underlying goal-directed behavior

**DOI:** 10.1016/j.neuron.2021.06.003

**Published:** 2021-08-04

**Authors:** Lisa Y. Duan, Nicole K. Horst, Stacey A.W. Cranmore, Naotaka Horiguchi, Rudolf N. Cardinal, Angela C. Roberts, Trevor W. Robbins

**Affiliations:** 1 Department of Psychology, University of Cambridge, Downing Street, Cambridge CB2 3EB, UK; 2 Department of Physiology, Development and Neuroscience, University of Cambridge, Downing Street, Cambridge CB2 3DY, UK; 3 Department of Psychiatry, University of Cambridge, Herchel Smith Building for Brain & Mind Sciences, Forvie Site, Robinson Way, Cambridge CB2 0SZ, UK; 4 Behavioural and Clinical Neuroscience Institute, Downing Street, University of Cambridge, Cambridge CB2 3EB, UK; 5 Cambridgeshire and Peterborough NHS Foundation Trust, Liaison Psychiatry Service, Box 190, Cambridge Biomedical Campus, Cambridge CB2 0QQ, UK

**Keywords:** goal-directed behavior, orbitofrontal cortex, ventromedial prefrontal cortex, anterior cingulate cortex, caudate nucleus, contingency degradation, obsessive-compulsive disorder, habits, common marmoset

## Abstract

Impaired detection of causal relationships between actions and their outcomes can lead to maladaptive behavior. However, causal roles of specific prefrontal cortex (PFC) sub-regions and the caudate nucleus in mediating such relationships in primates are unclear. We inactivated and overactivated five PFC sub-regions, reversibly and pharmacologically: areas 24 (perigenual anterior cingulate cortex), 32 (medial PFC), 11 (anterior orbitofrontal cortex, OFC), 14 (rostral ventromedial PFC/medial OFC), and 14-25 (caudal ventromedial PFC) and the anteromedial caudate to examine their role in expressing learned action-outcome contingencies using a contingency degradation paradigm in marmoset monkeys. Area 24 or caudate inactivation impaired the response to contingency change, while area 11 inactivation enhanced it, and inactivation of areas 14, 32, or 14-25 had no effect. Overactivation of areas 11 and 24 impaired this response. These findings demonstrate the distinct roles of PFC sub-regions in goal-directed behavior and illuminate the candidate neurobehavioral substrates of psychiatric disorders, including obsessive-compulsive disorder.

## Highlights

• Monkey pgACC-24 is necessary for detecting causal control of actions over outcomes
• Its projection target in the caudate nucleus is also implicated
• Three other subregions of the ventromedial prefrontal cortex are not necessary
• Anterior OFC-11 may mediate Pavlovian influences on goal-directed behavior

## Teaser

Duan et al. provide causal evidence in non-human primates that only PFC sub-region pgACC-24 and its target region in the caudate nucleus, but not mPFC-32, anterior OFC-11, rostral vmPFC-14, or caudal vmPFC-14-25, are critical for adapting instrumental responding following degradation of action-outcome contingencies in control of goal-directed behavior.

## Introduction

In everyday life, we continually make decisions based on our goals or go on “autopilot” to get through the day. Normal behaviors can either be goal directed, when performing an action to obtain a specific goal, or habitual, when a stimulus can trigger a well-learned response, regardless of its consequences. The goal-directed system is needed to adapt and remain flexible to changing environments and goals, whereas the habit system reduces cognitive load. However, an excessively dominant habit system can be maladaptive in some circumstances. Problems in the coordination and competition between the goal-directed and habitual systems are seen both in health (Balleine and O’Doherty, 2010; de Wit and Dickinson, 2009; Dolan and Dayan, 2013; Verhoeven and de Wit, 2018) and in neuropsychiatric disorders such as obsessive-compulsive disorder (OCD) (Gillan and Robbins, 2014; Robbins et al., 2019) and addiction (Ersche et al., 2016; Everitt and Robbins, 2005). Thus, understanding the neurobiological basis of goal-directed behaviors will provide insight into the etiology of such disorders.

To choose optimally, one needs to predict or believe that one’s action will cause the desired outcome (de Wit and Dickinson, 2009; Heyes and Dickinson, 1990). Whether an outcome (O) is contingent upon an action (A) depends not only on the probability (P) of the outcome occurring following the action (P(A|O)) but also on the probability of the outcome occurring in the absence of that action (P (A|¬O)). One’s sensitivity to changes in action-outcome (A-O) contingencies can be measured using a test of contingency degradation (Balleine and Dickinson, 1998; Dickinson and Weiskrantz, 1985; Hammond, 1980; Rescorla, 1966, 1968). Persistent responding following degradation of A-O contingencies implies residual habitual control (Balleine and O’Doherty, 2010). Contingency degradation assesses beliefs about the causal nature of contingencies and complements the use of “outcome devaluation” (which tests whether the desire for a goal drives actions) to measure goal-directed behavior (Heyes and Dickinson, 1990).

Evidence from human, non-human primate, and rodent studies has identified candidate neural systems for goal-directed behavior in mediating A-O contingencies within sub-regions of the prefrontal cortex (PFC), including ventromedial PFC (vmPFC), medial PFC (mPFC), orbitofrontal cortex (OFC), and the anterior cingulate cortex (ACC), as well as the caudate nucleus subcortically (reviewed in Balleine and O’Doherty, 2010). In human functional magnetic resonance imaging (fMRI) studies, sectors of vmPFC and anterior caudate were more active when subjects’ actions were highly predictive of the outcome than when their actions did not predict the outcome well (i.e., relating to P(A|O)) (Liljeholm et al., 2011; Tanaka et al., 2008). Studies of humans with vmPFC damage reveal intact learning of A-O contingencies but reduced awareness of such relationships under certain conditions (O’Callaghan et al., 2019; Reber et al., 2017). The vmPFC, however, is a large, heterogeneous region (Roberts and Clarke, 2019) comprising cytoarchitectonically (and likely functionally) distinct regions that cannot easily be resolved with fMRI and cannot be distinguished in human lesion studies.

Localized intervention studies in non-human animals have shed light on the causal role of PFC sub-regions in goal-directed actions. In non-human primates, non-specific ablations of dorsal ACC in macaques impaired their ability to adapt their actions to changes in outcome probabilities (Chudasama et al., 2013; Kennerley et al., 2006; Rudebeck et al., 2008). More selective excitotoxic lesions of area 32 (mPFC) or area 11 (anterior OFC [antOFC]) in marmosets impaired the initial acquisition of A-O contingencies, reflected in their subsequent insensitivity to contingency degradation (Jackson et al., 2016). Similar insensitivity has been described in rats with excitotoxic lesions of the prelimbic cortex (PL; variously labeled mPFC/vmPFC), lateral OFC, and the posterior dorsomedial striatum (DMS) (Balleine and Dickinson, 1998; Corbit and Balleine, 2003; Ostlund and Balleine, 2007; Yin et al., 2005). In contrast, anterior medial OFC lesions had no effect on this task, while affecting outcome devaluation (Bradfield et al., 2015). Thus, while it is clear that altered activity across a number of regions within prefrontal and cingulate cortices in humans and non-human animals can affect goal-directed behavior, differences in the test paradigms used and the extent of cross-species homology in prefrontal function hamper translation of the findings (Laubach et al., 2018; Roberts, 2020). For example, recent studies of vmPFC function in marmosets have highlighted functional discrepancies between this region in rodents versus primates (Roberts and Clarke, 2019; Wallis et al., 2017). Consequently, the present study provides a comprehensive comparison of the causal contribution of perigenual ACC, vmPFC, and OFC to goal-directed behavior, as measured by the sensitivity to contingency degradation in a New World primate, the common marmoset.

The structural organization of the marmoset PFC bears a greater resemblance to that of humans than to rodents (Burman and Rosa, 2009; Roberts et al., 2007; Vogt et al., 2013) and hence provides an important bridge between rodent and human studies. Moreover, the animal studies cited above mainly used contingency degradation to assess whether initial learning was goal directed or habit based, but not the subjects’ ability to respond to changes in A-O contingencies in established goal-directed behavior. We modified the classic rodent task (Balleine and Dickinson, 1998) and used a within-subjects design to ensure that animals were already exhibiting goal-directed actions before repeated acute manipulations in distinct brain regions. We examined the contribution of five prefrontal and cingulate sub-regions: areas 32 (mPFC), 24 (perigenual ACC), the boundary between areas 14 and 25 (area 14-25; caudal vmPFC), 11 (antOFC), and 14 (rostral vmPFC/mOFC) using temporary pharmacological inactivation via local microinfusion of a combination of a GABA_A_ agonist (muscimol) and a GABA_B_ agonist (baclofen) (mus/bac). As inactivation of pre-genual area 24 disrupted the sensitivity to contingency degradation and this region sends dense projections into the anteromedial caudate nucleus, we inactivated the caudate, using the glutamate receptor antagonist CNQX. Because positron emission tomography (PET) and resting-state fMRI have revealed OFC and ACC to be overactive in OCD patients, which may underlie the deficits in goal-directed behavior seen in this disorder (Robbins et al., 2019), including impaired knowledge of A-O contingencies in contingency degradation (Vaghi et al., 2019), we also determined the effects of the overactivation of prefrontal areas in marmosets. We achieved this via a glutamate reuptake inhibitor, dihydrokainic acid (DHK), which increases the extracellular levels of glutamate and enhances the excitability of cortical areas (Alexander et al., 2019; Bechtholt-Gompf et al., 2010; Muñoz et al., 1987). We define this operationally as overactivation because of the increased excitability and evidence of enhanced post-synaptic action of synaptically released glutamate by DHK (Muñoz et al., 1987).

## Results

### Novel procedure established marmosets’ sensitivity to contingency degradation

**Figure 1:**
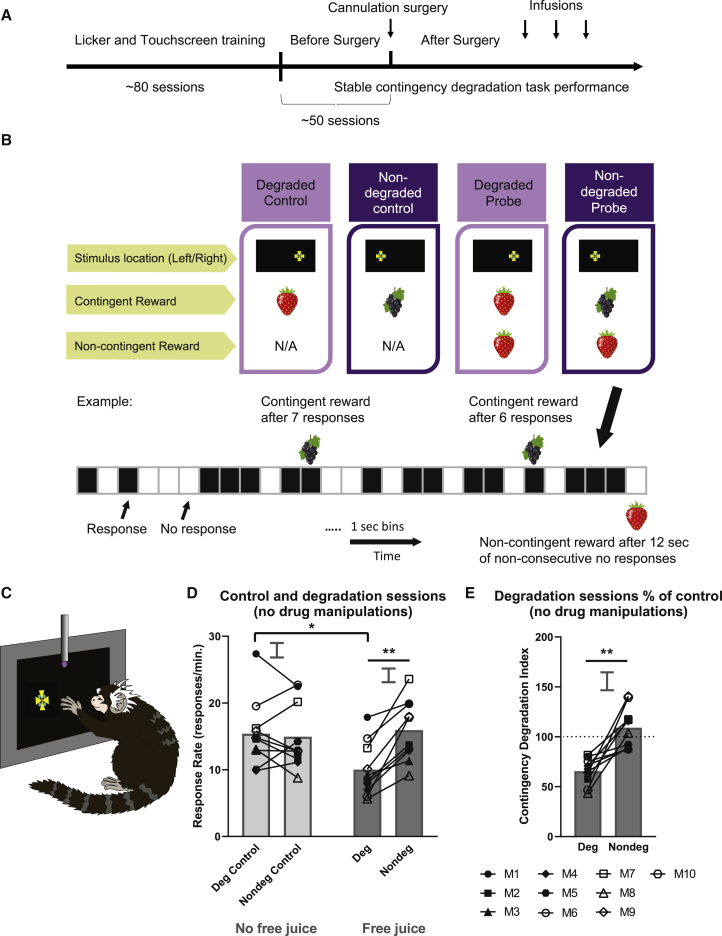
Experimental outline and a novel procedure to establish the sensitivity of marmosets to contingency degradation (A) Timeline of the experiment. Marmosets were pre-trained to engage with the testing apparatus and the reward being delivered from the licking spout before being given touchscreen training (see STAR Methods for more detail). Drug manipulations were conducted after sensitivity to contingency degradation had been established. (B) The novel contingency degradation task was divided into 4-day blocks. The first 2 days were control sessions and the subsequent 2 days were the contingency degradation probe sessions. In this figure, the example of degraded reward was strawberry juice and the non-degraded reward was blackcurrant juice. In the degraded probe session, the response-contingent reward (strawberry juice; example reward delivery probability = 0.1) was the same as the response non-contingent, “free” reward (strawberry juice; example probability = 0.067). In the non-degraded probe session, the response contingent reward (blackcurrant juice; example probability = 0.1) was not the same as the response non-contingent, free reward (strawberry juice; example probability = 0.067). Reward delivery probability was determined by dividing the 12-min session into 1-s bins. Black boxes indicate a response and white boxes a non-response within that 1-s bin. See STAR Methods for more details. (C) The marmoset touches the Maltese cross stimulus on the left and the associated juice reward can be retrieved from the licking spout situated in front of the touchscreen, according to a pre-programmed delivery schedule and session type. (D) The presence of free reward only affected marmoset responding when it was the same as the contingent reward (i.e., when the action-outcome [A-O] contingency was degraded) (free juice presence × degradation: F_1,4_ = 12.744, p = 0.0234). No difference in response rate was observed between the degraded control and non-degraded control (absence of free juice; p = 0.971). There was no significant difference when comparing non-degraded sessions with degraded controls (p = 0.954) and non-degraded controls (p = 0.677). (E) Marmosets were sensitive to changes in A-O contingencies. Marmosets were goal directed in that they showed a decrease in responding to the degraded session compared with the non-degraded session (p = 0.0009). Relevant graphs show the standard error of the differences between means (2 × SED) for degraded versus non-degraded comparisons, appropriate for post hoc pairwise comparisons. Deg, degraded. Nondeg, non-degraded. M, monkey. ^∗∗^p < 0.01, ^∗∗∗^p < 0.001.

Before the intra-cerebral infusions, we established that marmosets behaved in a goal-directed manner (Figure 1A). Marmosets were trained to associate each of two actions with a different outcome (juice rewards; Figure 1B; Table S1). The contingency degradation task was divided into 4-day blocks of test sessions. On weeks when there were no degradation probe sessions, animals received baseline control sessions across the 4 days. On weeks when there were degradation probe sessions (a minimum of every other week but often less frequently than that), the first 2 days were control sessions in which animals responded under a variable ratio schedule separately on independent sessions for each of the two rewards (degraded control or nondegraded control). Days 3 and 4 were contingency degradation probe sessions in which the A-O contingencies were modified such that one of the action-reward associations was degraded, whereas the other one was not (degraded probe or non-degraded probe). The marmosets were considered to behave in a goal-directed manner if they reduced their responding following the degradation of one of the A-O associations (i.e., when the juice reward was provided “freely” without the requirement of an instrumental response, and the response-contingent juice reward was the same juice) compared with when it was not degraded (i.e., when “free” and response-contingent rewards were different; Figure 1D). Importantly, marmosets only altered their responding when the A-O associations were degraded (same juice reward presented freely) when compared with sessions in which there were no free rewards (Figure 1D). Together, these results indicated that the reduction in responding was not simply due to the presence of free rewards (nor juice preference or availability of a variety of juices), but because of the weakening of perceived causality between a specific action and outcome.

We also provide in Figure S1 time courses of response rates over the sessions (same sessions as presented in Figure 1D) that show an accelerated decline in response rate relatively early in the session, in the degraded condition only. Inspection of individual curves in these sessions shows that the response rates of the animals, while individually variable, nevertheless conform to the general pattern seen in the group data.

To quantify sensitivity to contingency degradation, we defined a contingency degradation index (CDI) that took into account the marked individual variability in response rates shown by marmosets (e.g., see Figures 1D and S1). The CDI was defined as the percentage of response rates in degradation probe sessions compared to control sessions and is used as the main behavioral measure in subsequent analysis (Figure 1E; see STAR Methods). A CDI significantly lower in the degraded session than in the non-degraded session was taken as an indication of goal-directed behavior.

**Figure 2:**
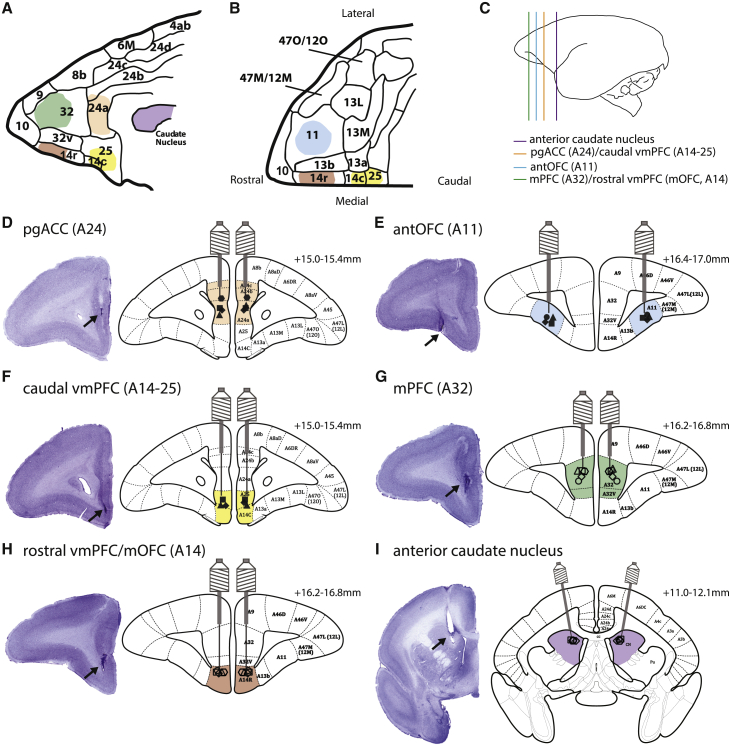
Schematic diagrams of cannulae placements in the PFC sub-regions and the caudate nucleus (A) Sagittal view of the medial surface of the PFC. Each color corresponds to a targeted brain region. (B) Ventral view of the OFC. (C) Target locations of pgACC (area 24), caudal vmPFC (area 14-25), antOFC (area 11), mPFC (area 32), rostral vmPFC/mOFC (area 14), and anterior caudate in relation to the whole brain. (D–F) Cannulae placements in areas 24 (D), 11 (E), and 14-25 (F). Area 14-25 was reached by vertically extending the area 24 injector, thus targeting both areas 24 and 14-25 via the same guide cannula. (G–I) Cannulae placements in areas 32 (G), 14 (H), and anterior caudate (I). Area 14 was reached by vertically extending the area 32 injector, thus targeting both areas 32 and 14 via the same cannula guide. Parcellation maps have been labeled based on Paxinos et al. (2012). See Table S4 for cannulation coordinates.

Marmosets (n = 10) then received drug manipulations in their respective cannulated brain regions (Table 1; see Table S2 for drugs and Table S3 for order of infusions) as depicted in Figure 2 (see Table S4 for cannulation coordinates).

**Table 1:** Cannulation locations for each marmoset.

Subject	Symbol	Area 11, n = 4	Area 24, n = 4	Area 14-25^a^, n = 3	Area 32, n = 5^b^	Area 14^a^, n = 5^b^	Caudate nucleus, n = 5^b^
M1	•	√ (12)	–	–	–	–	–
M2	▪	√ (12)	√ (12)	√ (12)	–	–	–
M3	▴	√ (8)	√ (12)	√ (12)	–	–	–
M4	♦	√ (12)	√ (12)	√ (12)	–	–	–
M5	⬣	–	√ (12)	–	–	–	–
M6	○	–	–	–	√ (12)	√ (12)	√ (8)
M7	□	–	–	–	√ (12)	√ (12)	√ (8)
M8	▵	–	–	–	√ (12)	√ (8)	√ (0)
M9	⋄	–	–	–	√ (12)	√ (12)	√ (8)
M10	⎔		–	–	√ (0)	√ (0)	√ (8)

M, monkey. Check marks indicate the brain regions that were targeted in that particular marmoset. Numbers in parentheses next to check marks are the number of infusions in each brain region, used to generate the data in this article. ^a^Areas 14-25 and 14 could be reached via extending the injectors through area 24 and area 32 vertical implants, respectively. ^b^n = 5 available, n = 4 collected data.

### Regional specificity of prefrontal drug effects on contingency degradation

**Figure 3:**
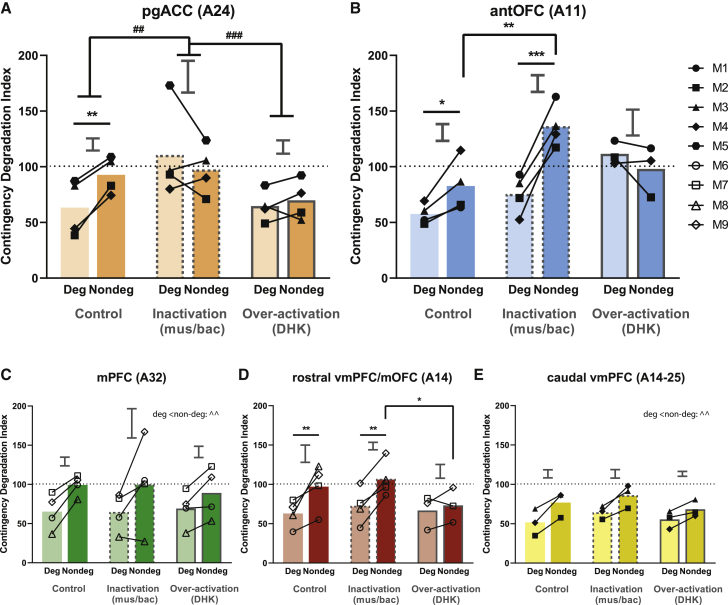
Effects of inactivation or overactivation of specific PFC sub-regions during contingency degradation (A) In area 24, inactivation and over-activation blunted the sensitivity of marmosets to contingency degradation (treatment × degradation: F_2,15_ = 4.429, p = 0.0308). There was a significant difference between degraded and non-degraded sessions only following saline infusion (p = 0.0065) but not after inactivation (p = 0.331) or overactivation (p = 0.601). This lack of difference after inactivation occurred due to a selective increase in responding in degraded sessions (p = 0.001) but not in non-degraded sessions (p = 0.912) when compared to saline. Responding across degraded and non-degraded sessions following overactivation was less than that of inactivation (p = 0.0005; see Figure 5A). (B) Area 11 (antOFC) inactivation apparently enhanced the sensitivity of marmosets to contingency degradation, while overactivation impaired it (treatment × degradation: F_2,13.287_ = 7.213, p = 0.00757). Marmoset responding in degraded sessions was significantly reduced, compared to non-degraded sessions, under both saline (p = 0.0407) and inactivation infusions (p = 0.0004), but no significant difference was observed after overactivation (p = 0.363). Further analysis revealed a significant increase in the difference in responding between degraded and non-degraded conditions after inactivation when compared to saline infusion (p = 0.0158). This effect was driven by a significant increase in responding in the non-degraded condition after inactivation when compared to saline (p = 0.0032) but not in the degraded condition (p = 0.248). (C) In area 32 (mPFC), marmoset responding in non-degraded sessions was significantly greater than that of degraded sessions across all treatment conditions (p = 0.0016). There were significant effects of degradation when saline data were considered alone (F_1,3_ = 21.176, p = 0.0193). (D) A significant difference between degraded and non-degraded sessions was observed following saline infusion (p = 0.0011) and inactivation (p = 0.0012) of area 14 (rostral vmPFC/mOFC). There were also significant effects of degradation when saline data were considered alone (F_1,3_ = 12.137, p = 0.04). Although no significant differences occurred between degraded and non-degraded sessions after overactivation (p = 0.445), this effect was most likely a non-specific drug effect (see Figure 5B). Responding during the non-degraded session after overactivation was significantly lower than that after inactivation (p = 0.0107) and trended lower than after saline (p = 0.0834). Conversely, the responding of marmosets during the degraded session after overactivation was not significantly different from that of inactivation (p = 0.848) or saline (p = 0.815). A similar pattern was observed in the baseline sessions, which tested the effects of drugs on marmoset responding without the presence of free rewards (see Figure 5B). (E) In area 14-25 (caudal vmPFC), marmoset responding in non-degraded sessions was significantly greater than that of degraded sessions across all drug conditions (p = 0.0016). There were significant effects of degradation when saline data were considered alone (F_1,2_ = 24.409, p = 0.0386). Relevant graphs show 2 × SED for degraded versus non-degraded comparisons (area 24: n = 4; area 11: n = 4; area 32: n = 4; area 14: n = 4; area 14-25: n = 3). Deg, degraded session. Nondeg, non-degraded session. Asterisk (^∗^) indicates a significant effect of the degradation × treatment interaction, # indicates a significant effect between treatments, ˆ indicates a significant effect between degradations. ^∗^/#/ˆp < 0.05, ^∗∗^/##/ˆˆp < 0.01, ^∗∗∗^/###/ˆˆˆp < 0.001.

Three-way ANOVA of the CDI in cohort 1 (areas 24, 14-25, 11) revealed a significant three-way interaction between treatment, degradation, and region (F_4,41.8_ = 5.3, p = 0.0016), which indicated that subsequent post hoc treatment × degradation effects for areas 24 and 11 were regionally specific (see Figure 3). This was further confirmed by permutation tests, (p = 0.002, 999 permutations). There was no such three-way interaction (F < 0.13, p = 0.88) for the second cohort of animals (areas 14 and 32, excluding caudate nucleus), also shown in Figure 3, and no effects of treatment or region on the degradation effect (F_2,31.05_ = 1.44, p = 0.25; F_1,31.05_ = 0.13, p = 0.72, respectively). However, there was a significant degradation effect (F_1,30.99_ = 17.81, p = 0.0002), a result also confirmed by a permutation test (p = 0.00008 with 50,000 permutations). There was no significant region × degradation interaction when comparing only the saline infusions across all brain regions (region × degradation: F_5,25.245_ = 0.122, p = 0.986).

### Disrupting activity in area 24 abolished the sensitivity of actions to contingency degradation

Following either inactivation or overactivation of area 24, the actions of marmosets were insensitive to degradation in A-O contingency (Figure 3A). Marmosets no longer distinguished between a reward that could only be obtained by performing an action (as in the non-degraded session) and one that could be obtained with or without an action (as in the degraded session). Their responding was the same regardless of whether the reward could be obtained freely. Although both inactivation and overactivation of area 24 blunted the sensitivity of marmosets to contingency degradation, their effects on responding differed: only the effects of inactivation were specific to contingency degradation. Overactivation of area 24 made the marmosets respond less, regardless of whether the A-O contingencies were degraded; this was likely due to DHK effects non-specific to contingency degradation, as such effects were also seen in baseline sessions in which no free reward was given (see STAR Methods for descriptions of baseline sessions and Figure 5A).

**Figure 4:**
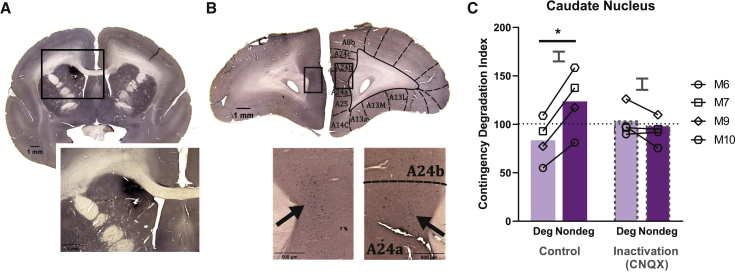
Inactivation of anterior caudate nucleus, which receives direct projection from area 24, impaired sensitivity to A-O contingencies (A) The retrograde tracer, cholera toxin B subunit, visualized in the left anterior caudate nucleus where it was injected. (B) Area 24, shown at the approximate placement used in this paper showing cell bodies of caudate projecting neurons within area 24. Ipsilateral projection from area 24 to the caudate is greater than that from the contralateral projection. (C) Inactivation of the caudate impaired sensitivity to contingency degradation. Significant treatment differences were observed on contingency degradation (treatment × degradation: F_1,9_ = 6.02, p = 0.0365). Inactivation (via CNQX) of the caudate nucleus that receives projection from the targeted area 24, resulted in a significant difference between degraded and non-degraded sessions following saline infusion (p = 0.0195), but not after inactivation (p = 0.543). Relevant graphs show 2 × SED for degraded versus non-degraded comparisons (n = 4). Deg, degraded session. Nondeg, non-degraded session. Asterisk indicates significant effect of degradation × treatment interaction. ^∗^p < 0.05.

**Figure 5:**
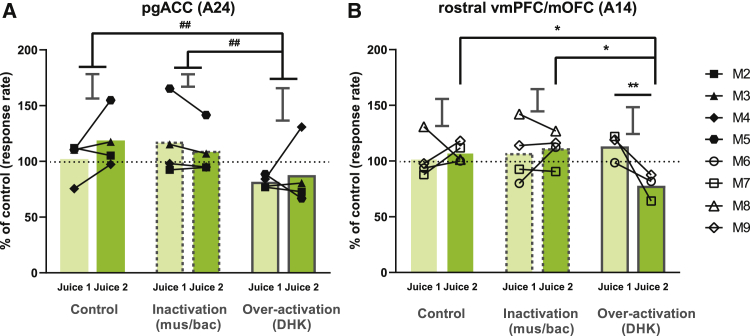
Effects of area 24 and 14 overactivation on baseline sessions (A) Overactivation of area 24 significantly affected responding compared to other manipulations (treatment: F_2,10_ = 14.846, p = 0.00102), where it significantly decreased responding across juice 1 and 2 when compared to inactivation (p = 0.00210) or saline (p = 0.00220). (B) Area 14 overactivation significantly affected responding in different juice conditions (juice condition × treatment: F_2,12.812_ = 6.358, p = 0.0121); overactivation specifically decreased responding to juice 2, which is the contingent reward in the non-degraded session in the contingency degradation task, compared to juice 1, which is the contingent reward in the degraded session in the contingency degradation task (p = 0.0038). Responding to juice 2 after overactivation was significantly lower than that following saline (p = 0.0202) or inactivation (p = 0.0232). Conversely, responding to juice 1 after overactivation was not significantly lower than that of saline (p = 0.330) or inactivation (p = 0.556). There was no significant difference in responding after overactivation between juice 2 in baseline sessions and the non-degraded session in the contingency degradation task (p = 0.651), whereas there was a significant difference between juice 1 and the degraded session in the contingency degradation task (p = 0.001). Relevant graphs show 2 × SED for juice 1 versus juice 2 comparisons (area 24, n = 4; area 14, n = 4). For baseline sessions of other brain regions, see Figure S5. Asterisk (^∗^) indicates significant effect of juice condition × treatment interaction, # indicates significant effect between treatments. ^∗^/#p < 0.05, ^∗∗^/##p < 0.01.

### Inactivation of area 11 enhanced, but overactivation blunted, sensitivity to contingency degradation

Inactivation of area 11 (antOFC) accentuated the difference in responding for a reward that was solely dependent on its availability through an action and one that was not (Figure 3B). This effect was driven by an increase in responding when the causal association between action and outcome was intact (non-degraded session) and not by a decrease in responding when the A-O association was weakened (degraded session). Correspondingly, the opposite effect was seen after the overactivation of area 11, which blunted the sensitivity of the animals to the degradation in A-O relationships (Figure 3B). This overactivation effect was unlike that observed for area 24 because it was not accompanied by an overall decline in responding (either in the degradation test or at baseline).

### Manipulations of areas 32, 14-25, or 14 had no specific effects on the sensitivity of actions to contingency degradation

Inactivation or overactivation of either area 32 (Figure 3C) or 14-25 (Figure 3E) did not affect responsivity to contingency degradation. Similarly, the inactivation of area 14 did not impair sensitivity to contingency degradation (Figure 3D). Although the overactivation of area 14 did blunt the contingency degradation effect (Figure 3D), the finding that it also reduced baseline responding suggests it was not specific to contingency degradation per se (see Figure 5B). Therefore, manipulations of areas 32, 14-25, and 14 appeared not to affect specifically the use of previously acquired response-outcome contingencies to guide responding following contingency degradation.

### Inactivation of the anterior caudate nucleus, the target region for area 24 projections, impaired sensitivity of actions to contingency degradation

After identifying area 24 as the main PFC sub-region necessary for detecting and acting upon changes in instrumental A-O contingencies (Figure 3A), we determined its target region within the caudate nucleus, as fronto-striatal pathways have been implicated in mediating goal-directed behavior (Balleine and O’Doherty, 2010). Rodent and macaque tracing studies had indicated that medial PFC and dorsal ACC project to the anterior dorsal striatum (Ferry et al., 2000; Haber et al., 1995; Heilbronner et al., 2016). However, tracing studies and the Marmoset Brain Connectivity Atlas have not previously investigated the connectivity of area 24a (i.e., the perigenual ACC [pgACC] targeted in the present study, with other brain regions [Majka et al., 2020; Roberts et al., 2007]; also see https://www.marmosetbrain.org/). Therefore, we infused a retrograde tracer, cholera toxin B subunit, into the anterior caudate via guide cannula in a marmoset not included in the behavioral data collection (Figure 4A). Cell bodies identified in area 24 confirmed the existence of an area 24-caudate pathway (Figure 4B). Bilateral projections from area 24 were observed, with greater ipsilateral projections (Figure 4B). In addition to area 24, the anterior caudate received PFC projections from areas 8 and 32 (area 32, see Figure S3B).

This region of the anterior caudate was then inactivated using CNQX (an AMPA glutamate receptor antagonist) to block excitatory glutamatergic input, including from the PFC, into this region (Galinanes et al., 2011; Darbin and Wichmann, 2008). Anterior caudate inactivation blocked the responsivity to contingency degradation (Figure 4C).

### Regional inactivations and overactivations in baseline sessions (without degradation) had differential effects on instrumental responding

Baseline sessions were conducted separately in close temporal proximity to the contingency degradation sessions, to examine the effect of pharmacological manipulations on baseline instrumental responding (see Figure S4 and STAR Methods for the task procedure of baseline sessions and further discussion of this control procedure). Overactivation of area 24 uniformly depressed responding when compared to saline or inactivation (Figure 5A), mirroring the effects in the contingency degradation task (Figure 3A). Saline infusions and inactivation of area 14 did not affect responding; however, overactivation of area 14 specifically depressed responding to the response-contingent reward (juice 2) in the non-degraded sessions of the contingency degradation task but not to the response-contingent reward (juice 1) in the degraded sessions (Figure 5B). This specific decrease in responding may explain the decline in responding after area 14 overactivation in the non-degraded session of the main contingency degradation task (Figure 3D).

Saline, inactivation, and overactivation of areas 11 or 14-25 or caudate nucleus did not alter responding during baseline sessions (Figures S5A, S5B, and S5D). Across all drug manipulations in area 32 (Figure S5C), marmosets increased responding to juice 2 compared to juice 1; this significant effect is likely non-specific arousal associated with infusions and handling since it occurred after all of the infusions, including saline. Why it should be selective to juice 2 may be because juice 2 was the designated preferred juice.

## Discussion

This study provides the first causal evidence in primates that perigenual area 24 is necessary for detecting and acting on changes in instrumental A-O contingencies and hence the capacity for understanding whether one’s behavior exerts control over the environment. Both inactivation and overactivation of perigenual area 24 impaired the response to contingency degradation, indicating that an optimal level or patterning of area 24 activity was required. Similar impairments were seen after reducing the excitatory input into that region of the caudate nucleus to which area 24 projects, indicating the potential involvement of a fronto-striatal circuit in exerting cognitive control over voluntary goal-directed behavior. In contrast, area 11 (antOFC) appeared to have an opposing influence, with inactivation enhancing and overactivation impairing contingency degradation. There were no effects of inactivation or overactivation of areas 32 (mPFC) or 14-25 (caudal vmPFC) on behavior following degradation. Inactivation of 14 (rostral vmPFC/mOFC) did not affect the response to contingency degradation either, while the blunting effect seen after overactivation was likely due to a non-specific drug effect rather than insensitivity to contingency degradation. The overall findings are summarized in Table 2.

**Table 2:** Results summary

Effect on sensitivity to contingency degradation	Degradation		Baseline	
	**Inactivation**	**Overactivation**	**Inactivation**	**Overactivation**

Area 24 (perigenual ACC)	Blunted	Blunted	No effect	Decreased across juice conditions
Area 11 (antOFC)	Enhanced	Blunted	No effect	No effect
Area 14-25 (caudal vmPFC)	No effect	No effect	No effect	No effect
Area 32 (mPFC)	No effect	No effect	Juice 2 > juice 1	Juice 2 > juice 1
Area 14 (rostral vmPFC/ mOFC)	No effect	Blunted^a^	No effect	Decreased in juice 2
Caudate nucleus (anteromedial)	Blunted	N/A	No effect	N/A

a This blunting of the sensitivity to contingency degradation may be due to a non-specific drug effect observed in the baseline sessions (see Results).

The vmPFC, which has been implicated in A-O contingency learning in human neuroimaging studies, subsumes a large heterogeneous region, including areas 10 and 14; ventral ACC regions 25, 32, and 24a; and very often in human lesion studies, orbitofrontal areas 11 and 13 (for reviews, see Roberts and Clarke, 2019; Schneider and Koenigs, 2017). Given this broad definition of vmPFC, it is not surprising that it has been implicated in a wide range of functions beyond A-O contingencies, including value comparison, reward processing, decision making, threat extinction, and social cognition (Hiser and Koenigs, 2018). Our results thus define, causally, specific regions of the primate vmPFC and mPFC that mediate the detection of changed A-O contingencies, resulting in altered expression of goal-directed behavior.

### Goal-directed control over responding requires optimal levels of activity in area 24, but not area 32

Perigenual ACC (area 24) is the only PFC sub-region in this study that disrupted animals’ sensitivity to the current A-O contingencies when either inactivated or overactivated. The impaired sensitivity after area 24 overactivation was nevertheless qualitatively different from that of inactivation. After area 24 inactivation, marmosets maintained their responding in the non-degraded session, but did not reduce responding in the degraded session (Figures 3A and S2A). However, after area 24 overactivation, while animals were impaired in differentiating their responses to degraded versus non-degraded sessions, they also decreased their responding uniformly across both sessions, potentially from other effects of the drug action (e.g., on general motivation, observed in baseline sessions, in which drug infusions were made without free rewards). The design of the experiment makes it very unlikely that these findings were artifacts of response competition, juice preference, juice variety, or juice satiety in the non-degraded condition. See Figures S2 and S6 for a detailed explanation.

This result is consistent with the previous literature showing that ACC mediates action-outcome learning and performance relevant to decision making (Chudasama et al., 2013; Hayden et al., 2009; Hayden and Platt, 2010; Holroyd and Coles, 2002; Holroyd and Yeung, 2012; Rushworth and Behrens, 2008; Rushworth et al., 2004, 2007; Wallis and Kennerley, 2011). Specifically, the firing rate of rostral ACC neurons tracks positive prediction error, unexpected reward delivery (Matsumoto et al., 2007), outcome surprisingness (unassigned prediction error), and likelihood of adjusting behavior (Hayden et al., 2011). The rostral ACC appears also to be important for selecting, maintaining, and integrating learned task information with multiple decision parameters across time and sessions (Amiez et al., 2006; Kennerley et al., 2006, 2011; Seo and Lee, 2007; Wallis and Kennerley, 2011).

In contrast to area 24, inactivation or overactivation of area 32 did not affect performance after contingency degradation; these findings are significant given previous rodent evidence that contingency degradation is impaired by PL lesions (Balleine and Dickinson, 1998; Corbit and Balleine, 2003). However, it is unclear how the rat PL relates to primate areas 24 and 32 (Heilbronner et al., 2016; Vogt et al., 2013), with the definition varying depending on criteria based on cytoarchitecture (Vogt et al., 2013), connectivity (Heilbronner et al., 2016), or function (Milad et al., 2007). The lack of involvement of area 32 in the current contingency degradation task stands in contrast to its role in the initial learning of A-O associations, shown via excitotoxic lesions of area 32 in the marmoset (Jackson et al., 2016). This is also consistent with evidence that excitotoxic PL lesions in rats impaired the acquisition of contingency learning (Balleine and Dickinson, 1998; Corbit and Balleine, 2003), if PL is homologous or analogous to area 32. While the role of area PL in the expression of goal-directed contingencies has yet to be investigated, such a dissociation is seen with respect to the sensitivity to outcome devaluation following PL lesions (Ostlund and Balleine, 2005; Tran-Tu-Yen et al., 2009). In contrast, area 24 (and not area 32) may be needed instead to express the effects of contingency degradation knowledge, highlighting a possible anterior-to-posterior transfer of information in the medial PFC. Tang et al. (2019) have suggested the boundary area of pregenual areas 24 and 32 to be a central hub for integrating information from sensory, motoric, limbic, and executive decision-making regions, based on anatomical connectivity patterns in macaques and humans.

### Excitatory projections from area 24 to the caudate nucleus affect behavioral expression of A-O contingencies

As predicted, inactivation of the anterior caudate blunting sensitivity to contingency degradation is consistent with studies showing that anterior caudate activity is involved in mediating A-O contingencies in humans (Liljeholm et al., 2011; Tanaka et al., 2008; Tricomi et al., 2004). In rats, the putative homolog of the caudate, the DMS, is also implicated in goal-directed behavior; in particular, findings have highlighted the posterior DMS (Hart et al., 2018a, 2018b; Yin et al., 2005), although there is increasing evidence for the involvement of anterior DMS (Corbit and Janak, 2010; Shipman et al., 2019). Thus, it can be hypothesized that a major output from area 24 for the expression of contingency degradation is to the anterior caudate. Of note, just as area 24 has been proposed as a connectivity hub (Tang et al., 2019), so too its striatal projection area, the rostral dorsal caudate, may serve to integrate inputs from other critical areas implicated in contingency knowledge (Liljeholm et al., 2011), including the OFC, ACC, and inferior parietal lobule (Choi et al., 2017). Given the mutual connections between areas 32 and 24 and their overlapping striatal projections to the anterior caudate (Averbeck et al., 2014; Draganski et al., 2008; Mailly et al., 2013; Roberts et al., 2007; Figure S3A), future studies should investigate the functions of this putative network in controlling the acquisition and expression of instrumental A-O contingencies.

### Areas 14/14-25 are not specifically implicated in the response to contingency degradation

Areas 14 (rostral vmPFC/mOFC) and 14-25 (caudal vmPFC) were not specifically involved in the response to changes in A-O contingencies. The complete lack of effects of inactivation is consistent with rodent studies, in which lesions of a putative homolog of these regions, the anterior mOFC, impaired the effects of outcome devaluation but not contingency degradation (Bradfield et al., 2015, 2018). Although marmosets receiving overactivation of area 14 did not differentiate between degraded and non-degraded sessions, the finding that baseline responding was also affected prevents any firm conclusions concerning contingency degradation. Consistent with a lack of involvement of area 14 in contingency degradation is the hypothesis that this region tracks and contrasts the intrinsic representations of action-associated outcome values during alternative choice situations (Noonan et al., 2010; Padoa-Schioppa and Assad, 2006; Rudebeck and Murray, 2011; Stalnaker et al., 2015; Valentin et al., 2007; Wallis and Kennerley, 2011). The decline in responding after area 14 overactivation in baseline sessions is consistent with the reported blunting of anticipatory arousal to high-value food reward in marmosets (Stawicka et al., 2020). Therefore, while area 24 could be important for mediating “causal beliefs” about behavior, area 14 may be more critical for comparative valuations in choice. Although imaging studies (Liljeholm et al., 2011; Tanaka et al., 2008) have shown a positive correlation between objective measures of causality and blood-oxygen-level-dependent (BOLD) activity within vmPFC, it is unclear whether this region is area 10 (Price, 2007) or area 14 (Mackey and Petrides, 2010).

### Inactivation of area 11 may enhance, and overactivation impair, expression of A-O associations, due to putative competition between Pavlovian (area 11) and instrumental (area 24) systems

Much evidence supports a role for OFC in acquiring and updating new information when tasks have strong Pavlovian components in both monkeys (Murray et al., 2007, 2015; Noonan et al., 2010; Rudebeck et al., 2008; Rushworth et al., 2007; Walton et al., 2010) and rats (Balleine et al., 2011; Ostlund and Balleine, 2007; Panayi and Killcross, 2018; Parkes et al., 2018). Although OFC impairments have been observed using stimulus-reinforcement learning tasks (Murray et al., 2007, 2015; Rolls, 2004), the OFC does not appear essential for the instrumental control of behavior (Ostlund and Balleine, 2007; Rudebeck et al., 2008; although see Gremel and Costa, 2013; Zimmermann et al., 2017). However, it has been implicated in mediating Pavlovian-to-instrumental transfer effects in rats (Cartoni et al., 2016; Holmes et al., 2010). In the present study, the inactivation of area 11 enhanced the effect of contingency degradation, whereas overactivation impaired it, suggesting that this region most likely exerts interfering Pavlovian control over instrumental responding. Specifically, in the current paradigm, instrumental responding to either the left or the right side of the touchscreen according to the specific A-O association (e.g., left: blackcurrant juice; right: strawberry juice) may be subject to interference by parallel Pavlovian approach responses, since the visual stimuli associated with each reward were identical (Figures 2B and 2C). Thus, the inactivation of area 11 may have reduced Pavlovian interference and hence enhanced instrumental performance, while overactivation produced the opposite effect (i.e., increased Pavlovian interference). In contrast, in our previous study of contingency degradation (Jackson et al., 2016), the distinct visual properties of the stimuli presented on the left or right differentially predicted the outcome and may thus have formed Pavlovian stimulus-outcome associations that facilitated performance. This may explain why OFC (area 11/13) lesions impaired contingency learning in that study.

### Methodological considerations, controls, and limitations

This study used an established method for inactivating cortical areas, using intracerebral infusions of a mixture of GABA_A_ and GABA_B_ receptor agonists. The possibility of diffusion from the site of infusion is relatively slight in relation to the volume of the different PFC regions, but in any case, the dissociable and selective nature of effects obtained suggests that such diffusion did not occur to any major extent. For rationale on using DHK for overactivation, see Introduction and STAR Methods.

### Implications

We show specific causal contributions of area 24 of the primate PFC to the detection and expression of A-O contingency changes as part of the control of goal-directed behavior. The persistence of responding during contingency degradation has been interpreted as an expression of habitual control (Balleine and Dickinson, 1998), although this is not necessarily the case (de Wit et al., 2018; Robbins and Costa, 2017), so further studies are required to establish whether area 24 exerts control over habits, in addition to goal-directed behavior. Contingency management can also be impaired following other PFC manipulations, such as overactivation of the anterior OFC (area 11) or area 24. These findings have implications for human psychiatric disorders such as OCD and schizophrenia, both of which involve impairments in goal-directed behavior (Barch and Dowd, 2010; Gillan et al., 2014; Morris et al., 2015). A recent study (Vaghi et al., 2019) found that OCD patients overresponded when response contingencies were manipulated to degrade the A-O contingency by providing “free” reinforcement as in the present study. OCD patients are known to have overactive regions of the PFC, notably the ACC and OFC (Baxter et al., 1988; Fitzgerald et al., 2011; Gillan and Robbins, 2014; Maia et al., 2008; Menzies et al., 2008; Pauls et al., 2014; Robbins et al., 2019; Whiteside et al., 2004), especially following symptom provocation (Nakao et al., 2005; Rauch et al., 1994). Our findings concerning the overactivation of both areas 24 and 11 are consistent with the pathophysiology of OCD and may indicate a possible role for maladaptive Pavlovian-to-instrumental transfer effects (Bradfield et al., 2017). Moreover, schizophrenia has been associated with a loss of GABAergic neurons in the anterior cingulate cortex (de Jonge et al., 2017); this may be associated with the impairments in goal-directed behavior seen in people with schizophrenia, which may underlie the “negative” symptoms of schizophrenia (Morris et al., 2018).

### Conclusions

Perigenual cingulate area 24 in the marmoset monkey is identified as a key cortical region in the detection and/or expression of changes in A-O contingencies. Other PFC regions, including anterior OFC (area 11), rostral (area 14), and caudal vmPFC (area 14-25) and area 32 in the mPFC, appear less involved, with inactivation of area 11 actually enhancing (and overactivation impairing) sensitivity to A-O contingencies. Our findings have implications for understanding the neural control of goal-directed behavior and for certain psychiatric disorders, including OCD and schizophrenia.

## STAR★Methods

### Key resources table

**Table:** 

REAGENT or RESOURCE	SOURCE	IDENTIFIER
**Antibodies**		

Rabbit polyclonal to c-fos primary antibody	Abcam	Cat #ab190289
Goat anti-Rabbit IgG H&L [Biotin] secondary antibody	Abcam	Cat #ab6720, RRID: AB_954902
Goat anti-choleragenoid primary antibody	Quadratech	703
Biotinylated donkey anti-goat secondary antibody	Stratech	bs-0294D-Biotin-BSS

**Chemicals, peptides, and recombinant proteins**		

Baclofen	Sigma Aldrich	Cat #B 5399, CAS 1134-47-0
CNQX disodium salt	Tocris	Cat #1045, CAS 79347-85-8
Dihydrokainic acid	Tocris	Cat #0111, CAS 52497-36-6
Muscimol	Sigma Aldrich	Cat #M1523, CAS 2763-96-4

**Deposited data**		

Raw data	This paper; Mendeley Data	http://dx.doi.org/10.17632/v75wdkr2f8.1

**Experimental models: Organisms/strains**		

Common marmoset (Callithrix jacchus)	University of Cambridge Marmoset Breeding Colony	N/A

**Software and algorithms**		

FIJI ImageJ	Schindelin et al., 2012	N/A
Ilastik	Berg et al., 2019	1.3.3
Illustrator	Adobe	CS6
Prism	GraphPad	8.3.0
R	R Development Core Team, 2020	3.5.1
Whisker	Cambridge University Technical Services Ltd.	4.6

### Resource availability

#### Lead contact

Requests for resources, reagents or questions about methods should be directed to Lead Contact, Lisa Y Duan (lisaduan33@gmail.com).

#### Materials availability

No new materials were generated in this study.

#### Data and code availability

Raw data from Figures 1, 3, 4, 5, S1, S2, S5, and S6 were deposited on Mendeley at https://dx.doi.org/10.17632/v75wdkr2f8.1. No new custom code, software, or algorithm was generated in this study.

### Experimental model and subject details

#### Common marmoset (*Callithrix jacchus*)

Ten common marmosets (*Callithrix jacchus*; four males and six females) were used for data collection for the contingency degradation task, while two marmosets (females) were used for tract tracing. All were experimentally naive at the start of the study. They were housed and bred on-site in a conventional barrier facility in the University of Cambridge Marmoset Breeding Colony. Experimental animals were housed in male-female pairs in custom-made housing (Tecniplast UK Ltd., Kettering, UK). The rooms were kept at a constant temperature of 24°C and relative humidity of 55%. The rooms were illuminated gradually from 7:00 am to 7:30 am and dimmed from 7:00 pm to 7:30 pm to simulate the day/night cycle. The marmosets were tested 4-5 days per week and not at the weekends. All monkeys were fed 20 g of MP. E1 primate diet (Special Diet Services) and sliced carrots five days a week after the daily behavioral testing session, with simultaneous free access to water for two hours. On weekends, their diet was supplemented with fruit, rusk, malt loaf, eggs, bread, and treats, and they had free access to water. The male marmosets were vasectomized to prevent pregnancy of their female partners. Their home cages were filled with environmental enrichment such as ropes and ladders. All animals were carefully monitored by the unit Named Animal Care and Welfare Officer (NACWO), researchers, the Named Veterinary Surgeon (NVS), and animal technicians. The projects were conducted under Home Office Project Licenses 70/7618 and P09631465, and all studies were verified and authorized by the unit NACWO. The projects were regulated under the Animals (Scientific Procedures) Act 1986 Amendment Regulations 2012 following ethical review by the University of Cambridge Animal Welfare and Ethical Review Body (AWERB).

### Method details

#### Behavioral testing apparatus and paradigm

##### Testing apparatus

Testing took place using an automated touch-screen apparatus (Biotronix, Cambridge, UK). Marmosets were transferred from their home cages to the testing apparatus via a transparent Perspex box, which is designed to be inserted directly into the testing apparatus for the duration of testing. The marmoset could move freely within the box and was not otherwise restrained. One side of the box was opened to enable the marmosets to interact with computer-controlled stimuli presented on a touchscreen (Campden Instruments, Loughborough, UK). They received liquid reinforcements from a spout/licker that was suspended centrally in front of the touchscreen (Figure 1C), which could deliver up to four different liquid rewards. The experiments were monitored and could be recorded by mounted cameras in the testing chamber. The MonkeyCantab program (R.N. Cardinal) controlled the touchscreen, pumps, spout and speakers via the Whisker control system (Cardinal and Aitken, 2010).

##### Licker and touchscreen training

The animals went through licker and touchscreen training before progressing to the contingency degradation task (Figure 1A). The main food reinforcer (banana milkshake, Nesquik) was initially introduced into the marmosets’ home cages and they were transferred to the testing apparatus for familiarization. They were shaped to approach the licking spout without experimenter guidance. The reward was delivered freely through the licking spout in the testing apparatus according to a fixed schedule: 8 s reward with 8 s inter-trial intervals (ITIs). During all reward delivery, an auditory cue (‘birdsong’) was also played. There were three phases of touchscreen training and each phase was completed in separate training sessions (Table S1). In the initial phase of touchscreen training, animals responded to a horizontal green bar that spanned the width of the touchscreen. Banana milkshake was delivered as a reward for 8 s. In the second phase, animals responded to a small green square in the center of the touchscreen. In the final training phase, the same green square was randomly presented to the left or right of the center of the touchscreen. After training on a fixed ratio 1 schedule, in which each response was reinforced, animals were switched to a variable ratio (VR) 3 schedule, in which they received a reward after every 2-4 responses. They then moved to a VR 6 schedule, and eventually a VR 10 (range 5-15 responses per reward) schedule. Following stable performance (3 consecutive sessions of consistent responding), the banana milkshake was replaced with blackcurrant, strawberry, summerfruit, or apple and mango juice. Each animal was assigned a pair of juices, with one juice always associated with the left stimulus, and the other the right stimulus. After another 3 stable sessions of performance, animals were transferred to the final contingency schedule (described in detail below) in which the green square was replaced with a compound, multi-colored stimulus (Maltese cross; Figure 1C). The sequence of touchscreen training is summarized in Table S1.

##### Contingency degradation task

The contingency degradation task measures goal-directed behavior (action-outcome associations). It used a four-day block design consisting of two control sessions followed by two contingency degradation probe sessions (Figure 1B). In the first two (control) sessions, animals responded to one of the stimuli (left or right location) for response-contingent reward in the first session, and the other stimulus on the opposite location for a different contingent reward in the second session. The two stimuli were identical and only differed in their display location (i.e., either on the left or the right of the center of the touchscreen, never displayed concurrently). Performance across the first two control sessions provided control data for comparison against the subsequent two additional degradation probe sessions. In the degradation probe session, the non-contingent, ‘free’ reward was introduced. In one session, the non-contingent reward was the same as the contingent reward, resulting in contingency degradation (degraded, action-outcome association weakened). In the second session, the non-contingent reward was the alternative reward not contingently available in that session, thus maintaining action-outcome associations for the contingent reward (non-degraded). The provision of the alternative juice on non-degraded sessions not only controls for any satiating effect of the freely provided juice in degradation sessions but also importantly controls for effects of response competition between approach to the free reward (licker) and instrumental touchscreen responding (Balleine and Dickinson, 1998).

To implement these degradation schedules, each 12-minute session was divided into 1 s bins. The mean probability of receiving the contingent reward was p = 0.1, i.e., an average of 10 responses would yield a reward (VR 10, range 5-15). Because of the large individual variance in response rate between marmosets, the probability of receiving the non-contingent reward was customized for each animal, and determined to ensure that they would detect the free rewards but not so many free rewards as to produce satiety and lead to the cessation of responding. For example, if the probability of non-contingent reward delivery is p = 0.067, then that means that animals received non-contingent reward, on average, every 15 s of non-responding (range 10-20 s).

Based on the contingency degradation hypothesis, animals that are sensitive to the action-outcome contingencies will show a much greater reduction in responding when the non-contingent juice is the same as the contingent juice (degraded condition) than when it is different (non-degraded condition). When assigning juices to marmosets we used a pair of juices that were relatively evenly matched for overall preference (Figure S6), but marmosets nevertheless very often show a mild preference. Therefore, for all manipulations where a marmoset showed a mild preference between juices, the preferred juice was always assigned to be the response-contingent juice in the non-degraded sessions, and the non-preferred juice was always the response-contingent juice in the degraded sessions (which is also the “free” juice). Because our measure of contingency degradation compares responding for a given juice in the degradation probe session (e.g., strawberry, in the presence of “free” juice) with its control session (e.g., strawberry, in the absence of “free juice”), all within a block, any slight juice preference will not influence any contingency degradation effect observed. In addition, the presence of the non-degraded condition rules out response competition and general juice satiety as an explanation for any effects that differentiate non-degraded from degraded conditions. Moreover, it should be noted that the small amounts of juice consumed by the animals during the test sessions (Figure S6) are grossly insufficient to produce general satiety per se.

##### Central infusions

Pharmacological manipulations of the brain occurred within subjects. They received infusions on the final two sessions of the contingency degradation probe sessions (degraded and non-degraded). In addition to the four-day contingency degradation block, a four-day baseline block was also conducted, which consisted of four control sessions (Figure S4). Marmosets received intracranial infusions on the final two sessions of that block, in which for one session they respond to receive Juice 1, the response-contingent reward used in the degraded sessions of the contingency degradation task, and the other session they receive Juice 2, the response-contingent reward used in the non-degraded sessions of the contingency degradation task. These control sessions enabled determination of the manipulation’s effects on baseline responding for reward, separate from any effects on responding mediated by changes in response contingencies. Thus, no free rewards were given in baseline sessions. The behavioral measure was calculated the same way as for the degradation sessions, without, of course, the need to account for free rewards (see below). These baseline sessions thus acted as a control for possible motivational and other non-specific sensorimotor influences of infused drugs on performance.

##### Pharmacological manipulations

For the prefrontal and cingulate brain regions there were three manipulations (saline, inactivation via muscimol/baclofen, over-activation via DHK) and for the caudate nucleus, there were two manipulations (saline and inactivation via CNQX). Whenever possible infusions were counterbalanced. Where a brain region was reached by extending the injector, the region above was always infused first, i.e., area 24 was infused before area 14-25 and area 32 was infused before area 14. Otherwise, where animals had cannulae in more than one brain region, infusions in brain regions were counterbalanced accordingly, i.e., area 11 was infused before or after area 24 and area 32 was infused before or after the caudate. Counterbalancing was also implemented with respect to whether (i) degradation sessions occurred before or after baseline and (ii) saline occurred before or after the experimental manipulation. Since the contingency degradation and baseline blocks consisted of 4 sessions, each block took place between Monday to Friday. Depending upon performance in the first two sessions of the block, in some weeks the marmosets just received control blocks with no infusions to ensure their performance was stable between infusion blocks.

The astrocytic excitatory amino acid transporter 2 (EAA2/GLT-1) inhibitor DHK (Anderson and Swanson, 2000; Arriza et al., 1994) has been shown to increase local concentrations of extracellular glutamate and to increase the excitability of the neuronal population and post-synaptic action as shown by microdialysis (Fallgren and Paulsen, 1996; Muñoz et al., 1987), electrophysiological recording (Muñoz et al., 1987), FDG-PET (Alexander et al., 2019) and immediate early gene *c-fos* expression (Alexander et al., 2019; Bechtholt-Gompf et al., 2010). We have referred to this DHK-induced state as ‘over-activation’, to simulate the relatively gross excitatory effects that occur in conditions such as OCD (see Introduction), and do not imply that DHK causes an enhancement of normal physiological activity by specific inputs to the region.

##### Behavioral measures

The main behavioral measure was the contingency degradation index (CDI). This was calculated in the degradation sessions as follows:

$$Contingency\;degradation\;index=\%\;of\;control\;session=\left(\frac{response\;rate\;in\;degraded\;or\;nondegraded\;session}{response\;rate\;in\;respective\;control\;session}\right)*100$$

This approach, measuring response rate as a percentage of that of the same subject in a control session, accounts for animals’ individual variability in response rate.

In the baseline sessions, an equivalent CDI-like measure was also used:

$$\%\;of\;control\;session=\left(\frac{response\;rate\;for\;Juice\;1\;or\;Juice\;2}{response\;rate\;in\;respective\;control\;session}\right)*100$$

During reward collection periods, animals did not have access to the touchscreen stimulus for responding. Thus, to control for the additional time animals spent drinking the free rewards during degradation probe sessions compared to control sessions, we calculated the index above using response rates (derived from non-reward collection periods), rather than absolute response numbers. This controls to a large degree for those periods when the animals are consuming the free rewards which necessarily prevent them from responding simultaneously to the touchscreen.

Hence, the response rate (responses per min.) in control sessions is calculated as follows:

$$Response\;rate=\frac{Response\;number}{\left(720-number\;of\;contingent\;rewards*10\right)/60}$$

Where 720 is session length in seconds and 10 is reward duration in seconds.

The response rate in degradation sessions:

$$Response\;rate=\frac{Response\;number}{\left[720-\left(number\;of\;contingent\;and\;noncontingent\;reward\right)*10\right]/60}$$

#### Cannulation procedure

Marmosets were premedicated with ketamine hydrochloride (Vetalar; 0.05 mL of a 100 mg/mL solution, i.m.; Amersham Biosciences and Upjohn, Crawley, UK) and then given a long-lasting nonsteroidal, anti-inflammatory analgesic (Carprieve; 0.03 mL of 50 mg/mL carprofen, s.c.; Pfizer, Kent, UK). They were intubated (using Intubeaze 20mg/ml lidocaine hydrochloride spray, Dechra Veterinary Products Ltd., Shropshire, UK), placed into a stereotaxic frame modified for the marmoset (David Kopf, Tujanga, CA) and maintained on 2.0%–2.5% isoflurane in 0.3 L/min O_2_ throughout the surgery. Heart rate, O_2_ saturation, respiratory rate, and CO_2_ saturation were all monitored by pulse oximetry and capnography (Microcap Handheld Capnograph, Oridion Capnography Inc., MA, USA) while core body temperature was monitored rectally (TES-1319 K-type digital thermometer, TES Electrical Electronic Corp., Taipei, Taiwan). Cannulae (Plastics One) were lowered bilaterally into desired brain regions using the stereotaxic arm. The coordinates for each brain region are listed in Table S4, and brain implant locations for each animal in Table 1. Coordinates were adjusted *in situ* where necessary based on cortical depth within the prefrontal cortex at +17.5 anteroposterior (AP), −1.5 lateromedial (LM) as previously reported (Roberts et al., 2007); this adjustment varied between −0.5 and −1.0mm. An extra depth check was performed for area 11 at its target AP and LM coordinates to obtain the target depth from the cortex. Each animal received bilateral cannulae in two target regions, areas 24 and 11, and area 32 and caudate nucleus. Access to area 14-25 or area 14 was via extended injectors through cannulae (vertically placed) in area 24 or area 32, respectively. Postoperatively, monkeys received the analgesic meloxicam (0.1 mL of a 1.5 mg/mL oral suspension; Boehringer Ingelheim, Germany) for the next 3 days as well as at least a full 7 days of “weekend diet” and water *ad libitum* to ensure complete recovery before returning to testing. The implants were cleaned with 70% ethanol during every infusion and at least once every week (and caps and cannula dummies changed) to ensure the cannula site remained free from infection.

#### Intracerebral drug infusion

The infusions were conducted using aseptic procedures. The injectors were connected to 10 μL syringes (Hamilton), which were mounted on an infusion pump. The marmoset was held comfortably by a researcher, the dust caps and dummy cannulae were removed, the guide cannulae were cleaned with 70% ethanol wipes. The injectors were placed into the guide cannulae, extending 1.5mm below the cannulae for areas 32, 1.0mm for area 24, area 11 and the caudate nucleus, 3.5mm for area 14 and 4.5mm for area 14-25. Bilateral infusions were carried out; for more information on the drugs infused, please see Table S2. Injectors were left in place for one additional minute for drugs to diffuse. The injectors were then taken out, dummy and caps replaced on the guide cannulae, and the marmoset was returned to the home cage.

#### Post-mortem histological processing

##### Assessment of cannula placement

At the end of the experiment, all monkeys were sedated with ketamine hydrochloride (Pharmacia and Upjohn, 0.05 mL of a 100 mg/mL solution, i.m.) and humanely euthanized with Euthatal (1 mL of a 200 mg/mL solution, pentobarbital sodium; Merial Animal Health Ltd; i.v.) before being perfused transcardially with 400 mL of 0.1 M phosphate-buffered saline (PBS), followed by 400 mL of 4% paraformaldehyde fixative solution over approximately 15 minutes. The cannulae and dental cement were carefully removed. After the brain was removed, it was left in the 4% paraformaldehyde fixative solution overnight, before being transferred to 0.01M PBS-azide solution for at least 48 hours and then transferred to 30% sucrose solution for a further 48 hours for cryoprotection. Brains were sectioned on a freezing microtome (coronal sections; 40-60mm), mounted on slides and stained with cresyl violet. The sections were viewed under a Leitz DMRD microscope (Leica Microsystems, Wetzlar, Germany). The cannula locations for each animal were represented on schematized coronal sections of the marmoset brain (Figure 2). Before euthanasia, some animals underwent infusions of drugs for *c-fos* verification and one animal underwent an anatomical tract-tracing study.

##### Tract tracer infusion, immunohistochemistry protocol and image analysis

The left caudate nucleus of one animal and the right area 24 of another animal (neither included in the behavioral study) was infused with the retrograde tracer cholera toxin B subunit (C9903, Sigma-Aldrich) via guide cannulae. The rate of infusion was 0.1 μL/min for 2 minutes, with 25 minutes of wait time for the drug to diffuse. The animals were perfused after 10 days and the brain was processed and cut. Each section was 40 μm thick, and one in every five sections was taken for immunohistochemistry. On day 1, the brain sections were put into well plates to wash three times for 10 minutes each in 0.1M Tris-NaCl (pre-made the day before, pH adjusted to 7.4; Tris-base, T4661-100 g, Sigma-Aldrich; NaCl – S7653-1Kg, Sigma-Aldrich). The washes occurred at room temperature and the wells were placed on a rocker. The 0.1M Tris-NaCl was changed between each wash in all situations. The sections were quenched to prevent endogenous peroxidase activity in 10% methanol and 10% H_2_O_2_ mixed solution for 5 minutes. The sections were then washed again three times for 10 minutes each in 0.1M Tris-NaCl. The sections were blocked in 0.1M Tris-NaCl with 0.2% Triton X-100 and 1% normal swine serum (S-4000, VectorLabs) for one hour at room temperature on a rocker. The sections were incubated overnight at room temperature, placed on a rocker, immersed in 0.1M Tris-NaCl with 0.2% Triton X-100, 1% normal swine serum and 1:2000 goat anti-choleragenoid primary antibody (703, Quadratech). On day 2, the brain sections were washed three times for 10 minutes each in 0.1M Tris-NaCl. They were then incubated for two hours at room temperature on a rocker, in 0.1M Tris-NaCl with 0.2% Triton X-100 and 1:200 biotinylated donkey anti-goat secondary antibody (bs-0294D-Biotin-BSS, Stratech). The brain sections were washed three times for 10 minutes each in 0.1M Tris-NaCl. They were incubated for 90 minutes at room temperature on a rocker with a ready-to-use avidin-biotin complex. The brain sections were washed three times for 10 minutes each in 0.1M Tris-NaCl. The sections were reacted with 3,3′-diaminobenzidine (DAB), using the ImmPactDAB horseradish peroxidase (HRP) Substrate Kit (SK-4100, Vector Labs). The reaction time inside DAB was determined empirically under the microscope. Once the desired staining was achieved, the section was immediately transferred to ice-cold 0.01M PBS to terminate the DAB reaction. The brain sections were mounted on gelatin-coated slides and dried overnight at room temperature. They were then dehydrated for 2 minutes each in solutions in the following order: 100% ddH_2_O, 25% ddH_2_O/75% ETOH, 100% ETOH, 50% ETOH/50% Xylene, 100% Xylene. The slides were coverslipped with DPX.

Images were acquired under bright field using a stereomicroscope (M205 FA; Leica, Wetzlar, Germany). Cell counting was conducted automatically using ilastik (version 1.3.3) (Berg et al., 2019) and FIJI (Schindelin et al., 2012).

### Quantification and statistical analysis

Data were analyzed using a mixed-model ANOVA using R version 3.5.1 (R Development Core Team, 2020). We used the lme4 package to conduct linear mixed-effects models with Type III analysis of variance and Satterthwaite’s method for degrees of freedom (Bates et al., 2015). Bartlett’s test was used to determine the homogeneity of variance. Initial 3-way ANOVAs were conducted on each of the two cohorts of marmosets: cohort 1 (areas 24, 11 and 14/25; Monkey M1-5) and cohort 2 (areas 14 and 32; Monkey M6-9) see Table 1. The caudate nucleus (cohort 2; Monkey M10) involved a different drug manipulation and so was analyzed separately. The factors were *region, treatment and degradation* (i.e., degraded versus non-degraded). A permutation analysis was also employed for cross-validation with fewer assumptions about the data using the permlmer function in the package predictmeans (Lee and Braun, 2012).

Subsequent analysis focused on each region separately. Each significant main effect (p < 0.05 adjusted for multiple testing using the multivariate t-distribution) was further examined using pairwise comparisons of least square means (lsmeans package in R) for specified factors in linear or mixed models. Fixed factors were the between-subject factor *infusion area* (region; area 11, area 24, area 14-25, area 32, area 14 and caudate nucleus) and the within-subject factors *treatment* (saline, mus/bac, DHK for PFC sub-regions; saline and CNQX for caudate nucleus) and *degradation* (degraded versus non-degraded). *Subject* was a random factor. To account for individual variabilities in response rate, the dependent variable was the contingency degradation index. Data for areas 24, 11 and 14-25 on degradation sessions were square-root transformed to satisfy the assumptions of the analysis of variance but the data presented in graphs are not transformed for comparison purposes. Data from drug manipulations on baseline sessions underwent the same analysis. We used the standard error of difference of the means (SED) as a more appropriate indication of the variance between means than the standard error of the mean (SEM), following ANOVA. The SED is calculated according to the equation given in Cochran and Cox (1957, p31).

Data from control and degradation sessions in the absence of manipulations (Figures 1D and 1E) were analyzed using within-subject repeated-measures ANOVA in R (afex package; R Development Core Team, 2020). Factors for the response rate data (Figure 1D) include two within-group factors of *degradation* (degraded versus non-degraded) and *free juice* (presence versus absence). Graphs were first completed in GraphPad Prism version 8.3.0 for Windows (GraphPad Software, La Jolla, California, USA), then transferred to Adobe Illustrator CS6 (Adobe Inc., San Jose, California, USA) for aesthetics.

## Supplemental information

Document S1. Figures S1–S6 and Tables S1–S4

Document S2. Article plus supplemental information
