# Supplementary material for: Controlling one’s world: Identification of sub-regions of primate PFC underlying goal-directed behavior
Source: Neuron. 2021 Aug 4;109(15):2485–2498.e5. doi: 10.1016/j.neuron.2021.06.003 (PMC8346232; doi:10.1016/j.neuron.2021.06.003)
Supplement: Document S1. Figures S1–S6 and Tables S1–S4 [file mmc1.pdf]

Neuron, Volume *109*

Supplemental information

Controlling one's world: Identification of sub-regions of primate PFC underlying goal-directed behavior

Lisa Y. Duan, Nicole K. Horst, Stacey A.W. Cranmore, Naotaka Horiguchi, Rudolf N. Cardinal, Angela C. Roberts, and Trevor W. Robbins

Supplemental
Figures

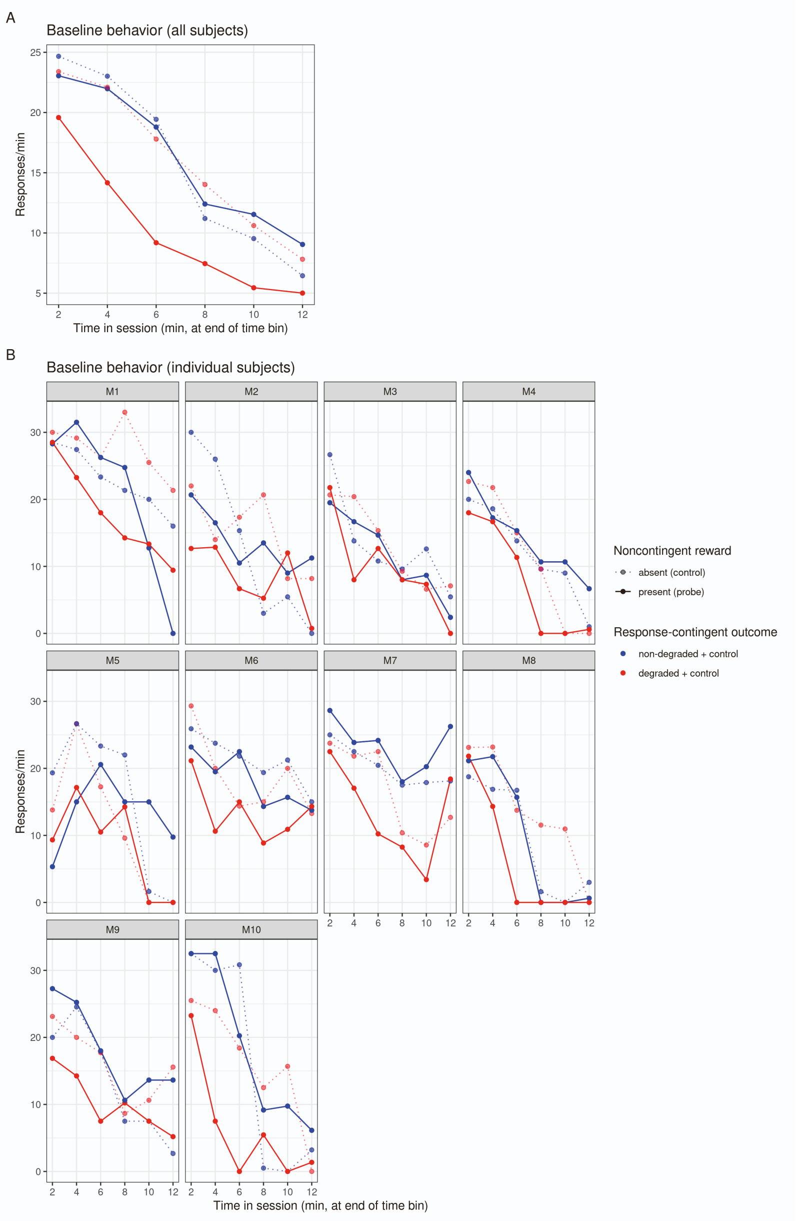

Figure S1. Time-courses of response rates over no-drug manipulation sessions (baseline behaviour). Related to Figure 1D. (A) All animals show early decline in their response rate in the degraded sessions only (red solid lines). We interpret this as relatively rapid learning about the contingency degradation manipulation, separate from the general decline in responding that occurs throughout all sessions, regardless of the availability of free juice or not. The latter we attribute to a more generalized reduction in motivational effort. (B) Response rate pattern of individual animals conform to the group data. The contingency degradation task specifically used in the current study can be understood as a paradigm measuring specifically (instrumental) contingency extinction; although unlike conventional extinction, there is no reward omission (Rescorla 1966, 1968). This is supported by consideration of the gradual reduction in responding during the degraded sessions (Figure S1, S2). Such response suppression is unlikely to be a passive 'unlearning' process, but like extinction, involves active inhibitory learning.

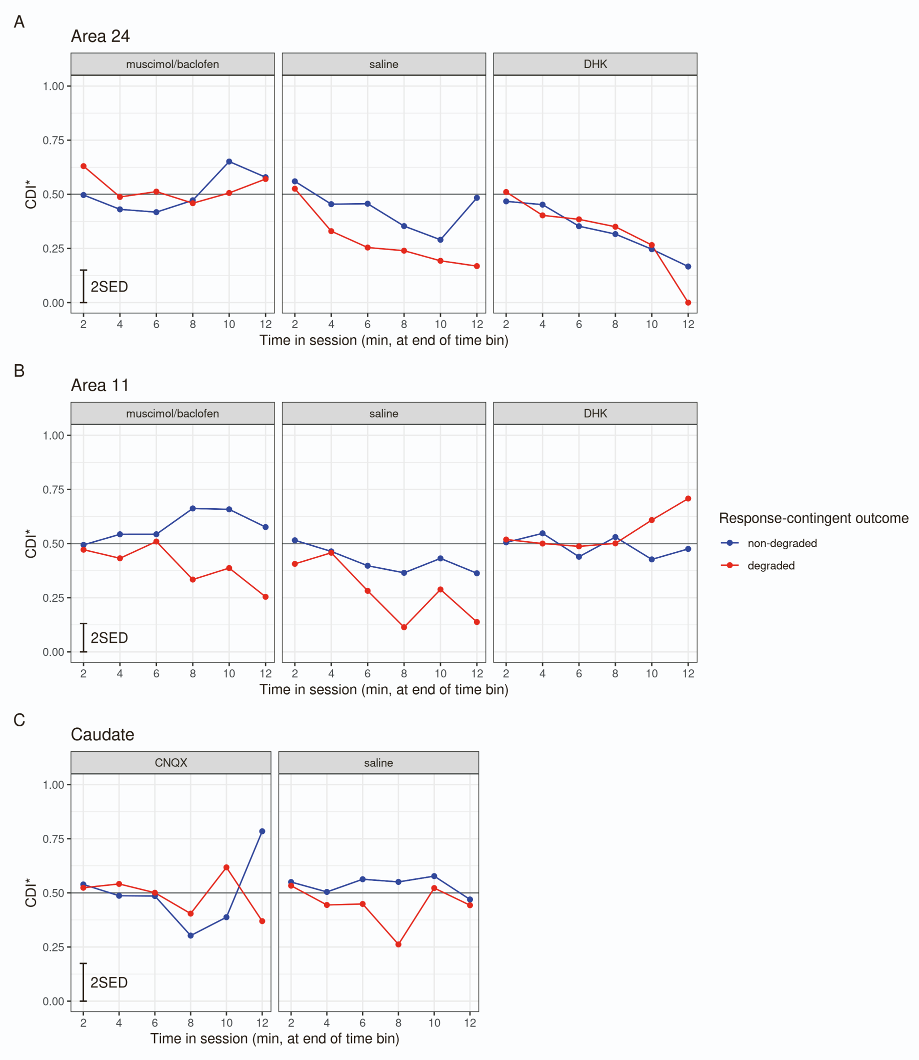

Figure S2. Time-courses of response rates over sessions that resulted in significant manipulation effect. Related to Figure 3 and 4. (A) Animals had lower responding in the degraded condition throughout test sessions, only after area 24 saline manipulation. Moreover, the finding that inactivation caused an increase in responding in the degraded condition compared to saline control infusions rules out any attribution of effects to satiation or the availability of another juice. This is less obvious though in the case of overactivation, where there was a reduction in responding on non-degraded conditions involving the availability of the second juice. It is possible, for example, that intra-area 24-DHK impairs taste discrimination (perception or preference) of the juices in this condition which makes it then equivalent to the degraded condition. However, the most parsimonious conclusion is that disruption of activity in area 24 impaired the response to contingency degradation. The computation of the instrumental contingency depends on combining two expressions, the probability of an outcome given an action and the probability of the outcome in the absence of an action (i.e. in context). Although one human neuroimaging study (Liljeholm et al., 2011) has suggested that these two computations and their product are mediated by different structures, it is not possible from the present results to determine which of these elements are mediated by area 24. However, it is evident that area 24 is implicated in the expression of altered contingent responding. (B) Animals had lower responding in the degraded condition throughout test sessions, after area 11 saline and inactivation, but not in over-activation. (C) Animals had lower responding in the degraded condition throughout test sessions, only after caudate nucleus saline manipulation.
CDI*: response rate of probe session / (response rate of probe session + response rate of control session)
Error bars for each brain region are twice the standard error of the difference (SED) for the degradation × time bin interaction from the analysis of data for that region (using treatment, degradation, and time bin as discrete predictors)

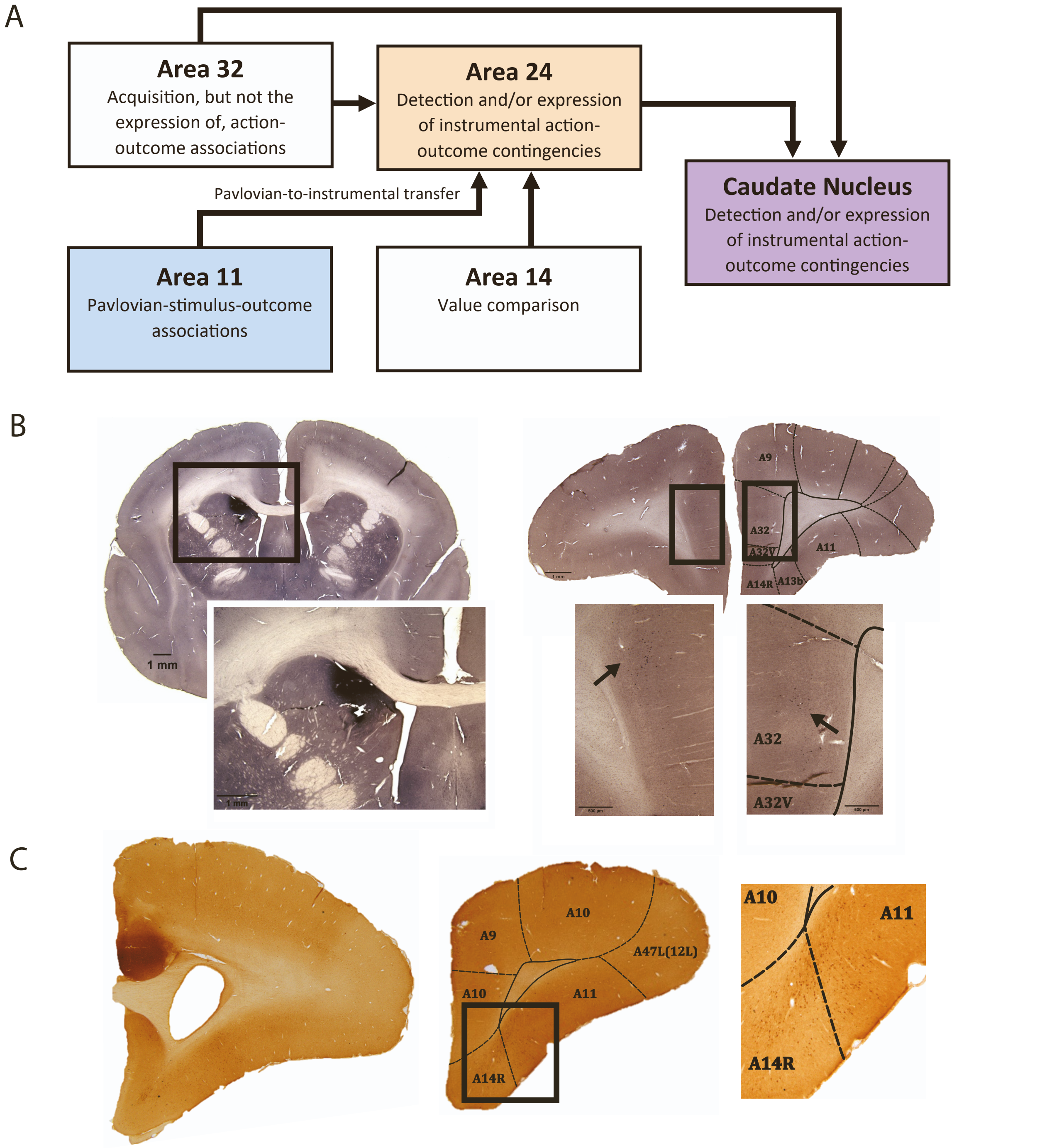

Figure S3. Cortico-cortico and fronto-striatal anatomical connectivity and contribution to goal-directed behavior on a circuit level. Related to Figure 3 and 4. (A) Proposed involvement of the four PFC sub-regions and caudate nucleus in goal-directed behavior. Black arrows indicate direction of neuronal projection verified in this paper. (B) Retrograde tracer injection site in the caudate nucleus (left) and associated cell bodies of projection neurons within area 32 (right). The caudate site is the same as that depicted in Figure 4. (C) Retrograde tracer injection site in area 24 (left) and associated cell bodies of projection neurons within area 11 and area 14 (middle- low power and right - high power). Parcellation maps have been labelled based on Paxinos et al. (2012).

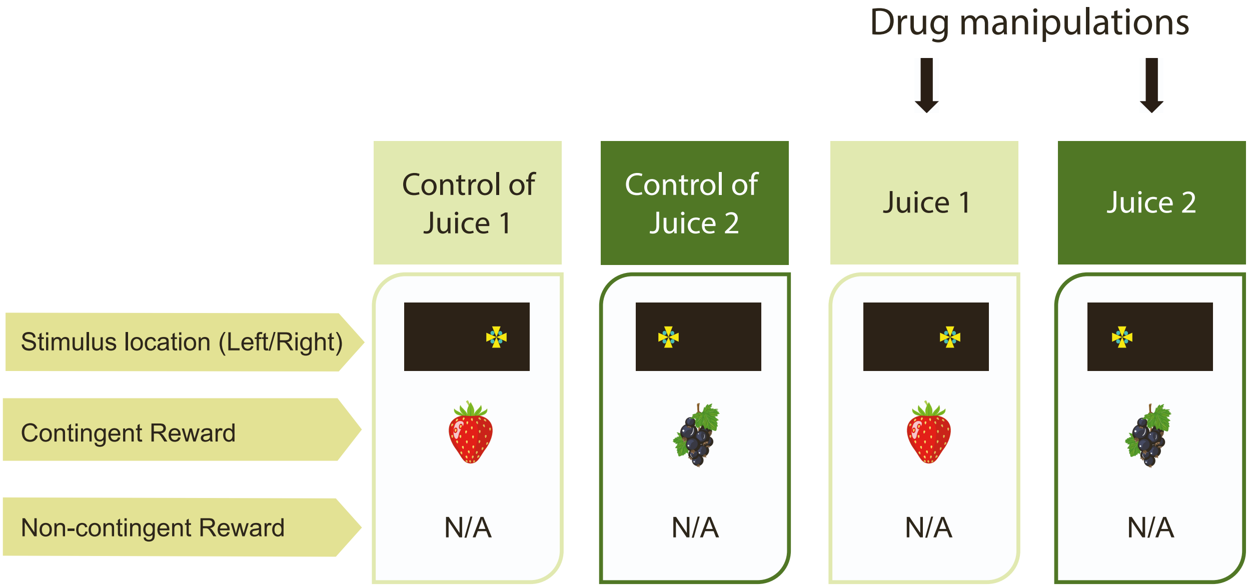

Figure S4. Task procedure for baseline sessions. Related to Figures 2, 5, S5 and STAR Methods. Baseline sessions are four-day blocks. In the first two days, marmosets respond to two action-outcome (A-O) associations on separate days, in which one of the A-O associations is going to be degraded and the other to not be degraded in the degradation sessions. The last two days are the same as the first two days but with marmosets receiving drug manipulations prior to testing. No free, non-contingent reward was present in any conditions. The only effects on baseline responding we observed were those of DHK (independent of responsivity to contingency degradation). Response rate decreases were seen for areas 24 and 14, the latter in one condition only, but in the case of area 24 these changes could not in themselves account for the impaired response to contingency degradation.

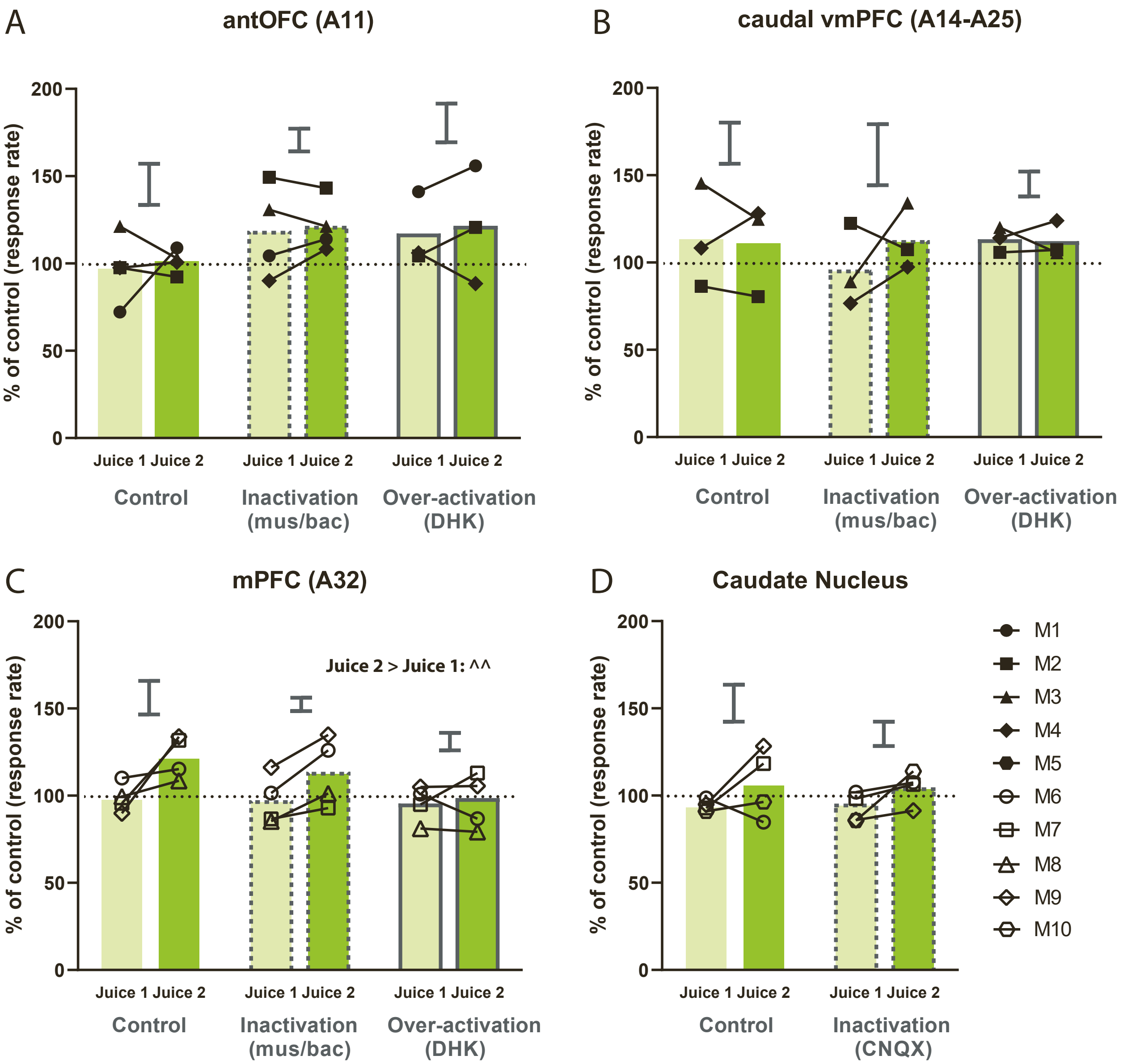

Figure S5. Effects of control, inactivation or over-activation of critical PFC and caudate nucleus regions in baseline sessions. Related to Figure 5. (A, B) Analysis of area 11 or area 14-25 baseline sessions revealed no main effects of juice conditions (area 11: $F_{1,\ 13.069} = 0.209$, $p = 0.655$; area 14-25: $F_{1,\ 10} = 0.245$, $p = 0.632$) or treatments (area 11: $F_{2,\ 13{,}684} = 2.684$, $p = 0.104$; area 14-25: $F_{2,\ 10} = 0.324$, $p = 0.731$). (C) For area 32, a main effect of juice conditions was observed ($F_{1,\ 15} = 9.338$, $p = 0.00801$), where marmosets significantly increased responding in Juice 2 when compared to Juice 1 across all drug manipulations ($p = 0.008$). (D) Analysis of caudate nucleus baseline sessions revealed no main effects of juice conditions ($F_{1,\ 12} = 4.084$, $p = 0.0662$) or treatments ($F_{1,\ 12} = 0.0696$, $p = 0.796$).

Relevant graphs show 2 X SED for "Jucie 1 v. Juice 2" comparisons (area 11: n = 4; area 32: n = 4; area 14-25: 3). ^ indicates significant effect between juice conditions. ^^: $p < 0.01$

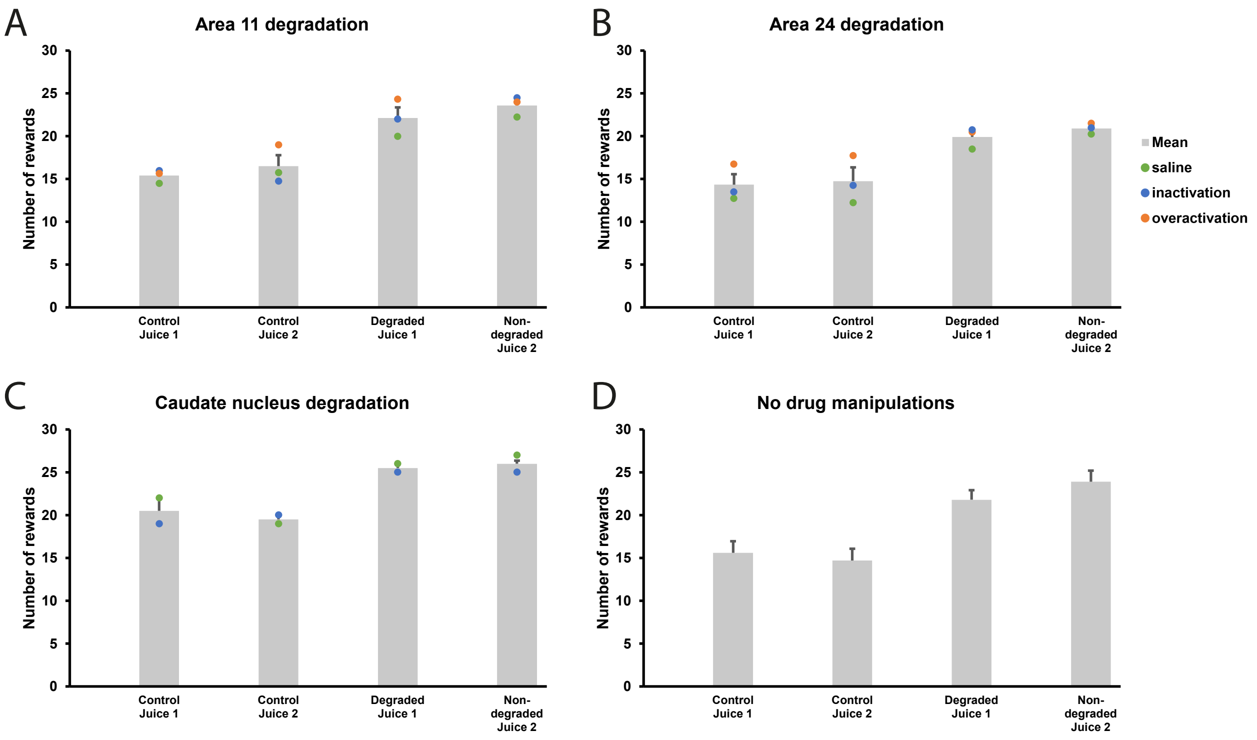

Figure S6. No difference between the number of Juice 1 and Juice 2 received in each session of a block (four-days) of contingency degradation sessions, within regions and between manipulations. Related to Figure 1, 3 and 4. (A-C) Within each brain region that had a manipulation effect on degradation (areas 11, 24 and caudate nucleus) animals received similar amount of Juice 1 and Juice 2, regardless of manipulation type. (D) In sessions where there were no drug manipulations (Figure 1D), animals also received similar amount of Juice 1 and Juice 2.

Tables

Schedule	Training Phase	Stimulus	Juice	Reward Length	ITI
FT	1	green bar	Banana milkshake	8 sec	1 sec
FT	2	green square centre	Banana milkshake	8 sec	1 sec
FT	3	green square L/R side	Banana milkshake	8 -> 5 sec	1 -> 3sec
FR1	3	green square L/R side	Banana milkshake	5 sec	-
VR 3	3	green square L/R side	Banana milkshake	5 sec	-
VR 6	3	green square L/R side	Banana milkshake	7.5 sec	-
VR 10	3	green square L/R side	Banana milkshake	10 sec	-
VR 10	3	green square L/R side	Juice	10 sec	-
Contingency	3	green square L/R side	Juice	10 sec	-
Contingency	3	Maltese cross L/R side	Juice	10 sec	-

Table S1. Touchscreen training schedule. Related to Figure 1 and STAR Methods.

Drug(s)	Mechanism	Concentration	Infusion Rate	Pre-Treatment Time	Source
Muscimol-Baclofen (Mus-Bac)	Muscimol: $GABA_A$ receptor antagonist<br>Baclofen: $GABA_B$ receptor antagonist	Muscimol: 0.1mM<br>Baclofen: 1.0mM	0.25μl/min for 2 mins	25 minutes	Sigma-Aldrich, St Louis, USA
Dihydrokainic acid (DHK)	Excitatory amino acid transporter-2 (EAAT2/GLT-1) inhibitor	6.25 nmol/μL	0.50μl/min for 2 mins	8-15 minutes	Tocris, Bristol, UK
CNQX	selective AMPA/Kainate receptor antagonist	1.0mM	0.3 μL/min for 1 min	8 minutes	Tocris, Bristol, UK

Table S2. Drugs used in the study. Related to Figures 3, 4, 5.

M1

Area	Treatment	Session type
11	sal	Degradation
11	m/b	Degradation
11	sal	Baseline
11	m/b	Baseline
11	DHK	Degradation
11	DHK	Baseline

M2

Area	Treatment	Session type
11	sal	Degradation
11	m/b	Degradation
24	sal	Degradation
24	m/b	Degradation
24	m/b	Baseline
24	sal	Baseline
11	m/b	Baseline
11	sal	Baseline
14-25	sal	Degradation
14-25	m/b	Degradation
14-25	sal	Baseline
14-25	m/b	Baseline
11	DHK	Degradation
11	DHK	Baseline
14-25	DHK	Baseline
24	DHK	Degradation
24	DHK	Baseline
14-25	DHK	Degradation

M3

Area	Treatment	Session type
11	sal	Degradation
11	m/b	Degradation
24	sal	Degradation
24	m/b	Degradation
24	m/b	Baseline
24	sal	Baseline
11	sal	Baseline
11	m/b	Baseline
14-25	sal	Degradation
14-25	m/b	Degradation
14-25	m/b	Baseline
14-25	sal	Baseline
14-25	DHK	Degradation
14-25	DHK	Baseline
24	DHK	Degradation
24	DHK	Baseline

M4

Area	Treatment	Session type
11	DHK	Degradation
11	Saline	Baseline
14-25	musbac	Degradation
14-25	Saline	Degradation
11	musbac	Degradation
11	Saline	Degradation
11	musbac	Baseline
14-25	Saline	Baseline
14-25	musbac	Baseline
11	DHK	Baseline
14-25	DHK	Baseline
14-25	DHK	Degradation
24	Saline	Degradation
24	musbac	Degradation
24	DHK	Degradation
24	Saline	Baseline
24	DHK	Baseline
24	musbac	Baseline

M5

Area	Treatment	Session type
24	m/b	Degradation
24	sal	Degradation
24	m/b	Baseline
24	sal	Baseline
24	DHK	Degradation
24	DHK	Baseline

M6

Area	Treatment	Session type
32	Saline	Baseline
32	Saline	Degradation
32	musbac	Baseline
32	musbac	Degradation
32	DHK	Baseline
Caudate	Saline	Baseline
Caudate	CNQX	Baseline
Caudate	Saline	Degradation
Caudate	CNQX	Degradation
32	DHK	Degradation
14	musbac	Degradation
14	Saline	Degradation
14	musbac	Baseline
14	Saline	Baseline
14	DHK	Degradation
14	DHK	Baseline

M7

Area	Treatment	Session type
32	musbac	Degradation
32	DHK	Degradation
32	Saline	Degradation
32	musbac	Baseline
32	Saline	Baseline
32	DHK	Baseline
14	DHK	Degradation
14	Saline	Degradation
14	musbac	Degradation
14	Saline	Baseline
14	musbac	Baseline
14	DHK	Baseline
Caudate	Saline	Degradation
Caudate	CNQX	Degradation
Caudate	Saline	Baseline
Caudate	CNQX	Baseline

M8

Area	Treatment	Session type
32	Saline	Degradation
32	DHK	Degradation
32	musbac	Degradation
14	Saline	Degradation
14	musbac	Degradation
14	Saline	Baseline
14	musbac	Baseline
32	Saline	Baseline
32	DHK	Baseline
32	musbac	Baseline

M9

Area	Treatment	Session type
32	DHK	Degradation
32	Saline	Degradation
32	musbac	Degradation
14	Saline	Degradation
14	DHK	Degradation
14	musbac	Degradation
Caudate	CNQX	Degradation
Caudate	Saline	Degradation
Caudate	CNQX	Baseline
32	Saline	Baseline
32	musbac	Baseline
32	DHK	Baseline
14	Saline	Baseline
14	musbac	Baseline
14	DHK	Baseline
Caudate	Saline	Baseline

M10

Area	Treatment	Session type
Caudate	Saline	Degradation
Caudate	CNQX	Degradation
Caudate	Saline	Baseline
Caudate	CNQX	Baseline

Table S3. Drug manipulation order received by each animal. Related to Figures 3, 4, 5.

Area	AP co-ordinate (mm)	LM co-ordinate (mm)	Depth (mm)
Area 11	+17.0	+/- 3.0	1.7 (from base)
Area 24*	+15.4	+/- 1.0	2.5 (from surface)
Area 32*	+16.8	+/- 1.0	1.5 (from surface)
Caudate	+11.0	+/- 2.2^	5.0^ (from surface)

Table S4. Cannulation co-ordinates. Related to Table 1. AP: anteroposterior; LM: lateromedial; *Area 14-25 and area 14 were reached by extending the injectors via the area 24 and area 32 guide cannulae, respectively. ^the caudate nucleus guide cannula was at 10 degrees angle away from the inter-aural line. Therefore, the LM of the guide entering the brain surface is +/- 3.2mm, whereas the actual targeted location inside the caudate nucleus is +/- 2.2mm and 5.0mm vertically from the brain surface.
